# Potential Cosmetic Applications of Dihydroartemisinin

**DOI:** 10.3390/molecules31020228

**Published:** 2026-01-09

**Authors:** Yifan Zhao, Mo Chen, Ying Zheng, Le Zhu, Cui Wu, Yue Ma, Ya Zhao, Dong Zhang, Haidong Jia, Lan Yang

**Affiliations:** 1 Artemisinin Research Center & Institute of Chinese Materia Medica, China Academy of Chinese Medical Sciences, Beijing 100700, China; yfzhao@icmm.ac.cn (Y.Z.); yma@icmm.ac.cn (Y.M.); lyang@icmm.ac.cn (L.Y.); 2 Shanghai Jahwa United Co., Ltd., Shanghai 200082, China; chenmo@jahwa.com.cn (M.C.); zhule@jahwa.com.cn (L.Z.); jiahaidong@jahwa.com.cn (H.J.); 3 College of Agroforestry and Medicine, The Open University of China, Beijing 100039, China; wuc@ouchn.edu.cn

**Keywords:** dihydroartemisinin, anti-aging activity, anti-hair loss effect, antibacterial activity, whitening activity, anti-glycation activity

## Abstract

In recent years, active monomers derived from Chinese herbal medicine and their derivatives have attracted significant attention in the field of skincare product development. Artemisinin and its derivatives, including dihydroartemisinin (DHA), exhibit diverse pharmacological activities such as anti-inflammatory, antibacterial, immunomodulatory, and antitumor effects, showing promising therapeutic potential in skin-related diseases. However, systematic studies on artemisinins in cosmetics are lacking. This study aimed to evaluate the cosmetic potential of DHA by investigating its anti-aging, anti-hair loss, antibacterial, whitening, and anti-glycation activities. Results showed that DHA exhibits multiple biological activities: DHA exhibits anti-aging activity by promoting collagen I synthesis in HDF cell, exhibits anti-hair loss effect by modulating VEGF and DKK1 expression in DPC cell, exhibits antibacterial activity against *Malassezia furfur*, exhibits whitening activity by suppressing melanin synthesis, and exhibits anti-glycation activity by suppressing glycation reactions. Overall, with the broad biological activities, we believe that DHA holds encouraging promise in the cosmetics industry.

## 1. Introduction

With the continuous improvement of people’s demands for the safety and effectiveness of cosmetics in today’s society, the innovative development of functional products has become a mainstream trend in the cosmetics industry. In China, the application of Chinese herbal medicine in the beauty industry has a history of several thousand years. Countless bioactive monomers and derivatives derived from traditional Chinese herbs have been proven to simultaneously regulate multiple skin-related signaling pathways, thereby demonstrating a variety of functions, such as anti-inflammatory, antioxidant, anti-aging, and skin-whitening effects. For example, ferulic acid can enhance skin elasticity and exhibits notable whitening, anti-inflammatory, smoothing, and moisturizing properties [1]. Thymol also has multiple functions, including antioxidant effects on the skin, anti-dermatitis properties, and the ability to facilitate wound healing [2]. Acetyl zingerone, a compound derived from the natural phytochemical zingerone, can alleviate photoaging by upregulating the gene expression associated with the Notch pathway and antioxidative stress response [3]. These reactive monomers and their derivatives show encouraging potential for the development of innovative skincare products and have thus become a key focus driving the future advancement of the cosmetics industry.

Artemisinin was a famous ingredient isolated from the Chinese materia medica Qinghao (*Artemisia annua* L.) by Professor Youyou Tu. As the WHO-recommended first-line antimalarial drugs, artemisinin and its derivatives (dihydroartemisinin, artemether, and artesunate) have saved millions of lives. Beyond the excellent antimalarial activity, artemisinin and its derivatives possess anti-inflammatory, antibacterial, immunomodulatory, antiviral and other effects, and play an important role in the treatment of skin diseases. The artemisinin derivative artesunate can significantly reduce dermatitis scores and decrease inflammatory cell infiltration in dinitrochlorobenzene induced atopic dermatitis (AD) model. In addition, it can inhibit the expression of pro-inflammatory factors such as IL-6, IL-17, IL-23, STAT3, and ROR-γt, while promoting the expression of anti-inflammatory factors such as transforming growth factor-β and suppressor of cytokine signaling-3 [4]. Artemether emulsion could significantly improve erythema, papule, and pustule scores in patients with rosacea. Compared with metronidazole emulsion, artemether emulsion showed higher safety and a lower recurrence rate [5]. In addition, artemisinins have the potential to improve seborrheic keratosis [6] and inhibit UVB-induced HaCaT cell senescence [7]. As a key bridge between artemisinin and the derivatives, dihydroartemisinin (DHA) was invented by Professor Youyou Tu in 1973. DHA exhibits excellent immunomodulatory and anti-inflammatory properties. Consequently, it holds significant applications in the treatment of psoriasis [8], lupus erythematosus [9], atopic dermatitis [10], and melanoma [11].

Our group is dedicated to discovery the bioactive components in *A. annua* L. and their potential applications in pharmaceuticals and cosmetics. Now, *Artemisia annua* L. has become a plant with high biological value and a potential source of cosmetic raw materials [12]. Our group have found that the *A. annua* L. extract holds potential for utilization in the treatment of eczema [13]. Maxim C. et al. found that *A. annua* extracts possess antioxidant capacity, supporting the potential use in dermatocosmetic formulations designed to protect the skin against oxidative stress [14]. Artemisinins have been reported to have beneficial effects in skin protection, including anti-fibrotic activity [15], alleviates skin lesions [16], and atopic dermatitis [17]. Currently, *A. annua* extracts and artemisinin have already been widely used as a cosmetic ingredient in products such as shampoos, face masks, and creams. However, as a semi-synthetic derivative of artemisinin, there have been relatively few reports on the research of DHA regarding its effectiveness in cosmetics. Herein, we conducted a series of activity evaluations on DHA, including anti-aging activity, anti-hair loss effect, antibacterial activity, whitening activity, and anti-glycation activity, based on the main functional characteristics of cosmetics. This study aims to explore DHA’s potential as a novel functional cosmetic additive and offer valuable insights for the development of new efficacy-driven cosmetic products.

## 2. Results and Discussion

### 2.1. DHA Exhibits Anti-Aging Activity by Promoting Collagen I Synthesis in HDF Cell

**Figure 1:**
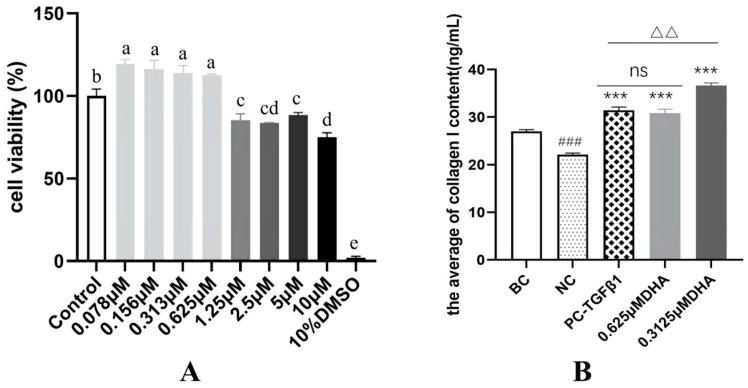
(**A**) Cellular viability. Data were presented as mean ± SD (*n* = 3). Different letters above bars indicate differences are significant at *p* < 0.05 (one-way ANOVA test and Tukey–Kramer post hoc test). (**B**) The contents of collagen I. Data were presented as mean ± SD (*n* = 3). BC: the blank control group treated with medium; NC: the model group treated with medium; PC-TGFβ1: the model group treated with TGF-β1; DHA: the model group treated with DHA. Compared to the BC group, ### *p* < 0.001; compared to the NC group, *** *p* < 0.001; compared to the PC-TGF-β1 group, ∆∆ *p* < 0.01; “ns”, not significant.

As shown in Figure 1A, at concentrations ranging from 0.078 μM to 0.625 μM, the cell viability was between 112.49% and 119.19%, indicating no cytotoxicity compared to the Control group and suggesting that DHA has the potential to promote HDF cell proliferation (*p* < 0.05). However, at higher concentrations of 1.25 μM to 10 μM, the HDF cell viability was significantly lower than those of the Control group (*p* < 0.05), indicating cytotoxicity. Therefore, the safe concentration range of DHA for human dermal fibroblasts is ≤0.625 μM. Based on the cytotoxicity results, two safe concentrations, 0.625 μM and 0.3125 μM, were selected to test the promotion of collagen I. As shown in Figure 1B, both 0.3125 μM DHA and 0.625 μM DHA significantly promoted the content of collagen I compared to the NC group (*p* < 0.01). Additionally, at the concentration of 0.3125 μM, the promotion was superior to the PC group (*p* < 0.01), with a promotion rate reaching 65.57%.

Collagen I is the most abundant collagen protein in the body. It accounts for 80–90% of the total collagen content in human skin and is vital in providing structural support to the skin, bones, and connective tissues. The age-related decrease in Collagen I has been linked to skin sagging and the development of wrinkles, making it a key focus in anti-aging treatments [18]. Our cytotoxicity assessment in HDF cell revealed that DHA is non-toxic within a specific concentration range (0.078 μM to 0.625 μM) and can even promote HDF proliferation. Furthermore, DHA (0.315 μM to 0.625 μM) significantly enhanced collagen I synthesis, surpassing the effects of the positive control (TGF-β1). Given that collagen I plays a crucial role in maintaining skin structure and elasticity, these findings suggest that DHA could be a valuable component in anti-aging formulation. Previous research showed that artesunate alleviated UVB-induced skin photoaging in HaCat cell [7]. These findings demonstrate the potential of artemisinins for anti-aging applications.

The exact mechanisms by which DHA promotes upregulation of collagen I remain to be elucidated, but several pathways may be involved. Firstly, given the central role of the TGF-β/Smad signaling pathway in regulating collagen synthesis in skin fibroblasts [19], DHA may influence collagen I production by modulating upstream signaling events associated with this pathway. In addition, DHA has reported anti-inflammatory activity [20], which could reduce inflammation-mediated extracellular matrix degradation and improve the microenvironment of HDF, thereby favoring the accumulation and maintenance of collagen I.

### 2.2. DHA Exhibits Anti-Hair Loss Effect by Modulating VEGF and DKK1 Expression in DPC Cell

**Figure 2:**
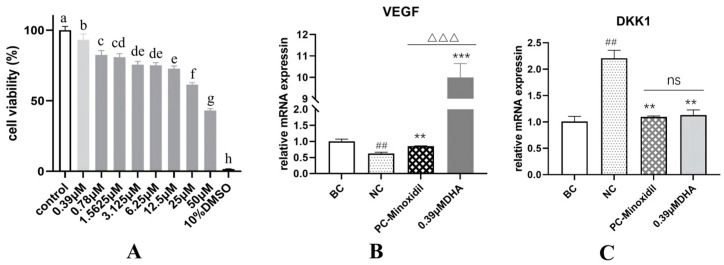
(**A**) Cellular viability. Data were presented as mean ± SD (*n* = 3). Different letters above bars indicate differences are significant at *p* < 0.05 (one-way ANOVA test and Tukey–Kramer post hoc test). The mRNA expression of VEGF (**B**) and DKK1 (**C**). Data were presented as mean ± SD (*n* = 3). BC: the blank control group treated with medium; NC: the model group treated with medium; PC-Minoxidil: the model group treated with Minoxidil; DHA: the model group treated with DHA. ## *p* < 0.01; compared to the NC group, ** *p* < 0.01, *** *p* < 0.001; compared to the PC-Minoxidil group, ∆∆∆ *p* < 0.001; “ns”, not significant.

As shown in Figure 2A, DHA at a concentration of 0.39 μM did not exhibit significant cytotoxicity, with cell viability at 93.07%. At other concentrations, cell viability was below 90%, indicating the presence of certain cytotoxicity. Therefore, 0.39 μM is considered the safe concentration of DHA for DPC cell. Based on the MTT cytotoxicity results, a safe DHA concentration of 0.39 μM was selected for the following tests. As shown in Figure 2, compared to the BC group, the relative mRNA expression was significantly reduced (*p* < 0.01), demonstrating the successful modeling. Compared to the NC group, DHA significantly promoted the relative mRNA expression of VEGF (*p* < 0.001) and was significantly superior to the PC-Minoxidil group (*p* < 0.001). At the same time, compared to the NC group, DHA significantly reduced the relative mRNA expression of DKK1 (*p* < 0.01), with no difference compared to the PC group (*p* > 0.05).

VEGF promotes the formation of new blood vessels, ensuring that hair follicles receive adequate oxygen and nutrients, which are crucial for their growth and survival. On the other hand, DKK1 (Dickkopf-1) regulates the Wnt signaling pathway, which is vital for hair follicle development and maintenance. By coordinating these processes, VEGF and DKK1 help maintain healthy hair follicles and prevent hair loss [21,22]. Notably, DHA significantly upregulated VEGF expression while downregulating DKK1 expression, indicating its potential to promote hair follicle health and prevent hair loss. The results suggest that DHA may serve as an effective alternative to conventional hair growth-promoting agents, such as minoxidil, providing a novel avenue for anti-hair loss treatments in cosmetic applications.

Mechanistically, DHA appears to exert anti-hair loss effects through the coordinated modulation of angiogenic and follicular signaling pathways. The upregulation of VEGF may enhance perifollicular angiogenesis and nutrient supply, while the downregulation of DKK1 alleviates inhibition of the Wnt/β-catenin pathway, thereby supporting hair follicle growth and maintenance [22,23].

### 2.3. DHA Exhibits Antibacterial Activity Against M. furfur

*Malassezia furfur* plays a significant role in skin health as a natural component of the skin’s microbiome. While it typically coexists harmlessly with the skin, an overgrowth of this yeast can lead to conditions like pityriasis versicolor, seborrheic dermatitis, and fungal folliculitis. These issues arise when factors such as excess oil production, immune system changes, or environmental conditions trigger the yeast’s overgrowth, resulting in atopic dermatitis, skin inflammation, rashes, and other symptoms [24,25]. Results showed that an inhibitory zone with a diameter greater than 9 mm indicates an inhibitory effect, while a diameter of 9 mm or less indicates no inhibitory effect. The negative control should show no inhibitory zone; otherwise, the experiment is invalid. Results showed that NC group (the diameter of antimicrobial-circle 7 mm) exhibited no inhibitory effects against *M. furfur*. And 0.5% OCT exhibited some antibacterial activity (22.13 ± 0.34 mm), and both 5 mM DHA (19.52 ± 0.61 mm) and 10 mM DHA (23.45 ± 0.45 mm) showed inhibitory effects against *M. furfur*. The lowest concentration of the antibacterial solution that completely inhibits colony growth is considered the MIC (Minimum Inhibitory Concentration) of the sample against *M. furfur*. The growth of a single colony can be disregarded. The MIC of DHA against *M. furfur* is about 4.5 mM, while the MIC of OCT solution against *M. furfur* is 2.5 mM. Briefly, our antimicrobial-circle test demonstrated that DHA exhibited significant inhibitory effects against *M. furfur*, a yeast associated with dandruff and other skin conditions. This suggests that DHA could be utilized in anti-dandruff shampoos or other skincare formulations aimed at controlling microbial imbalances on the skin and scalp.

Previous studies have shown that artemisinins can inhibit *Candida albicans* hyphal development [26] and disrupt mitochondrial energy metabolism in *Saccharomyces cerevisiae* by interfering with cellular metabolism [27]. Therefore, it is hypothesized that DHA may exert antimicrobial effects against *M. furfur* by disrupting its energy metabolism or cellular metabolic homeostasis.

### 2.4. DHA Exhibits Whitening Activity by Suppressing Melanin Synthesis

Melanin is a natural pigment found in the skin, hair, and eyes, responsible for their color and providing protection against UV radiation. However, its excessive production can lead to pigmentation. Skin pigmentation is primarily determined by three key processes: the synthesis of melanin within melanocytes, the transfer of melanosomes to keratinocytes, and the degradation of melanosomes [28]. Changes in melanin content are the most direct indicators of whitening efficacy [29].

**Figure 3:**
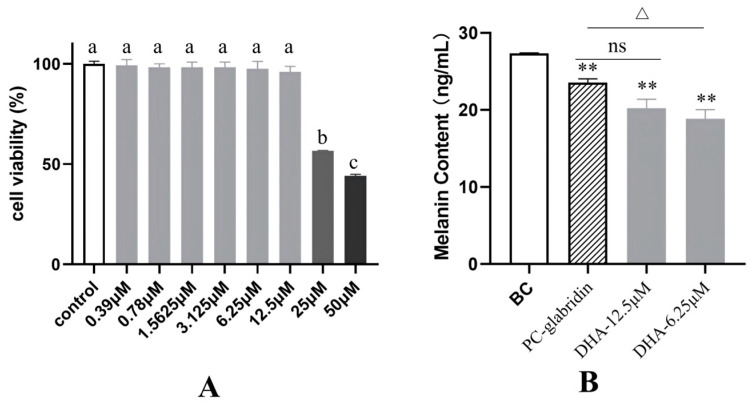
(**A**) Cellular viability. Data were presented as mean ± SD (*n* = 3). Different letters above bars indicate differences are significant at *p* < 0.05 (one-way ANOVA test and Tukey–Kramer post hoc test). (**B**) The melanin contents. Data were presented as mean ± SD (*n* = 3). BC: the blank control group treated with medium; PC-glabridin: the model group treated with glabridin; DHA: the model group treated with DHA. Compared with BC group, ** *p* < 0.01; compared to PC-glabridin group, ∆ *p* < 0.05 and ns means not significant.

DHA’s impact on melanocyte viability and melanin synthesis was evaluated. The results of the cell viability assay indicated that DHA did not exhibit cytotoxicity at concentrations ranging from 0.39 μM to 12.5 μM. Therefore, the safe concentration range of DHA for human melanocytes is ≤12.5 μM (Figure 3A). The inhibitory effect of DHA on melanin production is present in Figure 3B. Compared with BC group, the PC-glabridin group showed a significant decrease in the melanin content in human melanocytes (*p* < 0.01), with an inhibition rate of 14.05%. The 6.25 μM and 12.5 μM DHA also significantly inhibited the melanin content (*p* < 0.01), with inhibition rates of 31.11% and 26.11%, respectively. When compared with the PC-glabridin group, DHA at 6.25 μM exhibited a significantly greater inhibitory effect on melanin production (*p* < 0.05). These results indicated that DHA can exert a skin-whitening effect by directly inhibiting melanin production in melanocytes, highlighting its potential application in skin-brightening formulations. Mechanistically, like glabridin and artemisinins, DHA may potentially reduce melanin synthesis by targeting tyrosinase [30] and multiple melanogenic pathways, including STAT3/NF-κB and Bak-mediated mitochondrial apoptosis [31].

### 2.5. DHA Exhibits Anti-Glycation Activity

Glycation is a key factor in skin aging, which contributes to the formation of stable advanced glycation end-products (AGEs) through the glycation reaction (Maillard reaction) between proteins or lipids and reducing sugars under non-enzymatic conditions [32,33]. The accumulation of AGEs can impair protein function, leading to a series of issues associated with aging and chronic diseases. In this experiment, the anti-glycation ability of DHA was evaluated via AGEs as an assessment indicator. As shown in Table 1, compared with control group, DHA did not show significant inhibitory effects at concentrations ranging from 6.25 μM to 50 μM, but there was a trend of increased inhibition rate with rising concentrations. At the higher concentration, 100 μM DHA exhibited a significant inhibitory effect, with an inhibition rate of 16.39% (*p* < 0.01). Our findings suggest that DHA effectively inhibits glycation, the potential mechanism of action may involve the inhibition of AGEs formation [34]. Further research is needed to reveal the precise mechanism of action.

DHA (C_15_H_24_O_5_, MW = 284) is presented as a white powder or colorless needle-like crystals with no distinctive odor, low toxicity. Our experimental results showed that DHA exhibited several biological activities, including anti-aging, anti-hair loss, antibacterial, whitening, and anti-glycation effects, indicating the potential for the development of multiple functions products. However, several limitations of the present study should be acknowledged. Although several cosmetic-relevant targets (e.g., collagen I, VEGF, and DKK1) were evaluated, it remains unclear whether the observed effects of DHA are direct or secondary, as the underlying molecular mechanisms were not experimentally validated. In addition, the biological activities of DHA were not quantitatively compared with those of established cosmetic actives, such as vitamin C, peptides or kojic acid. Further mechanistic investigations and comparative efficacy studies are therefore needed to substantiate the cosmetic relevance of DHA. Furthermore, the use of DHA in cosmetic formulations presents several challenges, including compliance with regulatory requirements, formulation compatibility at effective concentrations, potential intellectual property considerations, and overall market acceptance. Firstly, regulatory approaches to drug ingredients vary across countries. For example, China generally prohibits the use of drug ingredients in cosmetic products, whereas in the United States and Japan, certain drug ingredients may be permitted in cosmetics, provided that they comply with applicable filing, registration, and regulatory management requirements. Secondly, due to its poor water solubility and limited stability, the incorporation of DHA into cosmetic formulations requires specific formulation optimization and protective strategies to ensure the effectivity and stability. Thirdly, DHA has been available for more than five decades, and potential intellectual property risks represent an important consideration that cannot be overlooked during its development as a cosmetic ingredient. Finally, as a pharmaceutical ingredient, DHA has seen limited application in cosmetic products; therefore, appropriate scientific communication and interpretation are necessary to improve public understanding and enhance market acceptance.

## 3. Materials and Methods

### 3.1. Chemicals, Reagents, and Cells

DHA was obtained from the Chongqing Wuling Mountain Pharmaceutical, Kunming Pharmaceutical Group (Chongqing, China, the batch number: C00220160402). Dulbecco’s modified Eagles’ medium (DMEM), Dulbecco’s Phosphate-Buffered Saline (DPBS), Mesenchymal Stem Cell Medium (MSCM), 0.25%Trypsin-0.02%EDTA, penicillin-streptomycin-neomycin antibiotic mixture (Cat: 15640) and Dulbecco’s Modified Eagle Medium: Nutrient Mixture F-12 (DF-12) were purchased from Gibco (Grand Island, NY, USA). PBS (pH 7.4, 220628-05) was purchased from Solarbio Life Sciences (Beijing, China). Fetal bovine serum was purchased from Royacel (Lanzhou, China). DMSO, 5α-dihydrotestosterone (DHT), H_2_O_2_, glucose and minoxidil were purchased from Sigma (Saint Louis, MO, USA). TGF-β1 was purchased from Pepro-Tech (Rocky Hill, CT, USA). The RNAiso Plus kit (Cat: AG21101), the Evo M-MLV RT Master Mix kit (Cat: AG11706), and the SYBR Green Pro Taq HS Premix kit (Cat: AG11701) were purchased from Accurate Biology (Changsha, China). Cortisol was obtained from MP Biomedicals (Santa Ana, CA, USA). Pure water was purchased from Watsons (Hong Kong, China). The primary human dermal fibroblast cell (Lot: Fb20081902), primary dermal papilla cell (Lot: 210727), and primary human melanocyte (Lot: MC210817) were purchased from Guangdong Biocell (Dongguan, China). *Malassezia furfur* ATCC44344 was purchased from Hua Yue Enerprises Holdings (Guangzhou, China). Glabridin (Cat: B20474) was obtained from Shanghai Yuanye Bio-Tech (Shanghai, China). Aminoguanidine hydrochloride (Cat: G1909194) was purchased from Aladdin Scientific (Shanghai, China). Octopirox was purchased from Yimingtai (Shandong) Biotechnology Co., Ltd. (Tai’an, China). The Human Collagen I ELISA kit (CSB-E08082h) was purchased from CUSABIO (Houston, TX, USA).

### 3.2. Evaluation of Anti-Aging Activity

#### 3.2.1. Cytotoxicity Test of DHA in Human Dermal Fibroblast (HDF) Cell

Cellular viability was assessed via the MTT assay, following the manufacturer’s instructions. Specifically, HDF cell were seeded into 96-well plates at a density of 8000 cells per well and incubated overnight at 37 °C in a humidified 5% CO_2_ atmosphere. The drug was administered when the cell confluence reached 40%~60%. The DHA groups were treated with 200 μL DHA solutions at concentrations ranging from 0.078 μM to 10 μM. The positive control (PC) group received 200 μL of MSCM supplemented with 10% DMSO, and the solvent control (Control) group was treated with 200 μL DMEM. All experiments were conducted in triplicate. After 24 h of incubation, the culture medium was carefully removed, and 200 μL of MTT reagent (0.5 mg/mL in PBS) was added to each well, followed by a 4 h incubation period. The MTT solution was subsequently aspirated, and 150 μL DMSO was introduced to dissolve the formazan crystals. Absorbance measurements were conducted at 490 nm with a BioTek Epoch Microplate Reader (Winooski, VT, USA).

#### 3.2.2. Assessment of Anti-Wrinkle Efficacy

The HDF cell was seeded in 24-well plates at 4 × 10^4^ cells per well and subsequently cultured overnight under standard conditions (37 °C, 5% CO_2_) until 40–60% confluency was achieved. The DHA groups were treated with 2 mL DHA solution (0.3125–0.625 μM), the positive control (PC-TGF-β1) group was treated with 2 mL 100 ng/mL TGF-β1 in DMEM, the negative control (NC) group and the blank control (BC) group were treated with 2 mL DMEM. After 24 h incubation, the test group, PC group and NC group were exposed to UVA radiation (Philip, The Netherlands) at 30 J/cm^2^ and then incubated at 37 °C, 5% CO_2_ for 24 h. After another 24 h incubation, the supernatant was collected and stored at −80 °C. The concentration of type I Collagen in the supernatant was quantified with a commercial Collagen I ELISA kit (Houston, TX, USA), following the instructions provided by the manufacturer.

### 3.3. Evaluation of Anti-Hair Loss Effect

#### 3.3.1. Cytotoxicity Test of DHA in Dermal Papilla Cells (DPC) Cells

Cellular viability was evaluated using the MTT method, following the manufacturer’s instructions. Specifically, DPC cell was seeded into 96-well plates at a density of 16,000 cells per well and incubated overnight at 37 °C in a humidified 5% CO_2_ atmosphere. The drug was administered when the cell confluence reached 60%. The DHA-treated groups received 200 μL of DHA solutions at concentrations ranging from 0.39 μM to 50 μM. For the positive control (PC), 200 μL of MSCM supplemented with 10% DMSO was applied, while the solvent control (Control) group was administrated 200 μL of MSCM alone. All experiments were conducted in triplicate. After 24 h of incubation, the medium was gently aspirated, and each well was supplemented with 200 μL of MTT reagent (0.5 mg/mL in PBS), followed by a 4 h incubation period. Subsequently, the MTT solution was discarded, and 150 μL of DMSO was introduced to dissolve the formazan crystals. Absorbance measurements were taken at 490 nm using a BioTek Epoch Microplate Reader (Winooski, VT, USA).

#### 3.3.2. Evaluation of the Anti-Hair Loss Effect of DHA in DHT-Induced DPC

The DPC cell was seeded in 6-well plates and then incubated at 37 °C, 5% CO_2_ overnight to achieve a confluency of 60%. The DHA group was treated with 2 mL MSCM containing 800 nM DHT and 0.39 μM DHA, the positive control (PC-Minoxidil) group was treated with 2 mL MSCM containing 800 nM DHT and 800 nM minoxidil, the negative control (NC) group was treated with 2 mL MSCM containing 800 nM DHT and the blank control (BC) group was treated with 2 mL MSCM. After 24 h incubation, DPC cell was washed twice with PBS and then moved to enzyme-free EP tubes. Total RNA isolation from the tissue was performed with the RNAiso Plus reagent, and the cDNA was subsequently generated using the Evo M-MLV RT Master Mix kit, adhering to the manufacturer’s instructions. The primers were commercially obtained from Accurate Biology Co., Ltd. (Changsha, China). The forward primer sequence of VEGF is 5′-TAAGTCCTGGAGCGTTCCCT-3′, and the reverse primer sequence is 5′-ACGCGAGTCTGTGTTTTTGC-3′. The forward primer sequence of DKK1 is 5′-ATGCGTCACGCTATGTGCTG-3′, and the reverse primer sequence is 5′-TGGAATACCCATCCAAGGTGCTA-3′. The quantitative reverse transcription polymerase chain reaction (qRT-PCR) was conducted with a Bio-Rad CFX96 Real-Time System (Hercules, CA, USA) and the SYBR Green Pro Taq HS Premix kit, following the instructions provided by the manufacturer. The 2^−ΔΔCT^ method was employed for data analysis, and the relative expression levels of target genes in experimental samples were expressed as fold changes compared to the blank control group (BC).

### 3.4. Evaluation of Antibacterial Activity

#### 3.4.1. Antimicrobial-Circle

*Malassezia furfur* was employed to assess the antimicrobial effect of DHA by well diffusion method. There are three groups: the DHA group, the negative control group (sterile saline) and the positive control group (0.5% OCT solution). Under aseptic conditions, a volume of 100 µL of the DHA sample (5 mM and 10 mM) were pipetted into wells on test plates containing a bacterial medium with a concentration of 10^5^–10^6^ CFU/mL. Cover the plate and incubate it in a constant-temperature incubator for 48 h. After incubation, measure the diameter of the inhibition zone and record the results.

#### 3.4.2. Minimum Inhibitory Concentration

There are three groups: the DHA group, the negative control group (sterile saline), and the positive control group (OCT solution). First, the bacterial suspension of the *M. furfur* was prepared and adjusted to a concentration of 10^7^–10^8^ CFU/mL using sterile saline. The DHA sample solution and OCT solution were then, respectively, diluted with sterile deionized water to obtain different concentrations and kept at room temperature. For each test, 2.5 mL of the sample solution was added to a tube containing 2.5 mL of bovine bile salt liquid medium and mixed thoroughly. Then, 100 μL of the bacterial suspension (10^7^–10^8^ CFU/mL) was inoculated into the tube.

All samples were incubated in a VORTEX-5 shaker (Haimen Kylin-Bell Lab Instruments Co., Ltd., Nantong, China) at 37 °C for 48 h. After incubation, 1 mL of liquid culture from each test tube was transferred onto a flat dish and poured into the culture medium for further incubation at 37 °C. The results were observed after another 48 h. The minimum inhibitory concentration (MIC) of the sample against *M. furfur* is defined as the lowest concentration at which colony growth is completely inhibited. The growth of a single colony is considered negligible.

### 3.5. Evaluation of Whitening Activity

#### 3.5.1. Cytotoxicity Test of DHA in Human Melanocytes

Cellular viability was assessed via the MTT assay, following the manufacturer’s instructions. Specifically, human melanocytes were seeded into 96-well plates at a density of 10,000 cells per well and incubated overnight at 37 °C in a humidified 5% CO_2_ atmosphere. The drug was administered when the cell confluence reached 40%~60%. Cells in the DHA-treated groups received 200 μL of DHA solutions at concentrations ranging from 0.39 μM to 50 μM. For the positive control (PC), a 200 μL volume of DF-12 medium supplemented with 10% DMSO was applied, whereas the solvent control (Control) group was administrated 200 μL of DF-12. All experiments were conducted in triplicate. After 24 h of incubation, the culture medium was carefully aspirated, and replaced with 200 μL of MTT reagent (0.5 mg/mL in PBS), followed by a 4 h incubation period. The MTT solution was discarded, and 150 μL of DMSO was introduced to solubilize the formazan crystals. Absorbance readings were taken at 490 nm with a BioTek Epoch Microplate Reader (USA).

#### 3.5.2. Melanin Content Test Based on Human Melanocytes

Human melanocytes were inoculated into 6-well plates at an inoculum density of 2 × 10^5^ cells/well and incubated overnight in an incubator (37 °C, 5% CO_2_). The DHA groups were treated with 2 mL DHA solution (6.25–12.5 μM), the positive control (PC-glabridin) group was treated with 2 mL glabridin solution (193 μM), the blank control (BC) group was treated with 2 mL medium. After 24 h incubation, the melanocytes were digested with 0.25% trypsin at 37 °C, collected in a 1.5 mL centrifuge tube, and centrifuged at 10,000 r/min for 10 min, and the supernatant was discarded. 1 mL of 1 mol/L NaOH aqueous solution containing 10% DMSO was added to the centrifuge tube and heated in an 80 °C water bath for 40 min. After thermal incubation, 200 μL of the supernatant was transferred to a 96-well plate, and the OD value at 405 nm was measured using a BioTek Epoch Microplate Reader (USA).

### 3.6. Evaluation of Anti-Glycation Activity

The glycation reaction solution (2×) containing 80 mg/mL bovine serum albumin and 240 mg/mL glucose was prepared using 0.1 mol/L PBS and filtered through a 0.22 μm filter membrane. Equal amounts of glycation reaction solution (2×) and DHA solution (6.25–100 μM) (DHA group) or 1% aminoguanidine hydrochloride solution (positive control) was mixed and labeled as sample group, and the fluorescence intensity was labeled as A. The sample solvent was used instead of glycation reaction solution (2×) in sample solvent group, and the fluorescence intensity was labeled as B. Equal amounts of PBS solution and glycation reaction solution (2×) were mixed, and the fluorescence intensity was labeled as C. Equal amounts of PBS solution and the solvent of glycation reaction solution (2×) (PBS) were mixed, and the fluorescence intensity was labeled as D (Table 2).

The mixture was homogenized and incubated in a DH400BII constant temperature incubator (Taiste, Tianjin, China) at 55 °C for 4 days. At the end of the reaction, the incubated solution was cooled to room temperature. And 200 μL of the glycation reaction solution (2×) was sequentially taken and added to the 96-well plate. The inhibition rate of advanced glycation end products (AGEs) was detected and calculated using a Microplate Reader (BioTek Epoch, USA) at 370 nm excitation wavelength and 440 nm emission wavelength. The calculation method is as follows: Inhibition rate (%) = [1 − (A − B)/(C − D)] × 100.

### 3.7. Statistical Analysis

Statistical analysis was conducted with SPSS software (version 26.0, Chicago, IL, USA), employing one way ANOVA analysis and the Student’s *t*-test. All graphical representations were created using GraphPad Prism software (version 8.0.2, San Diego, CA, USA).

## 4. Conclusions

In this study, we comprehensively evaluated the biological activities of DHA and its potential applications in the cosmetics industry. Results indicated that DHA exhibited certain biological activities: anti-aging activity, anti-hair loss effect, antibacterial activity, whitening activity, and anti-glycation activity. These characteristics make DHA a promising candidate for inclusion in skincare and haircare applications. Future research should focus on the molecular mechanisms, formulation optimization, clinical trials and potential synergistic effects with other cosmetic ingredients to fully realize DHA’s potential applications in cosmetics.

## Figures and Tables

**Table 1:** Results of AGEs inhibition rate.

Sample	Group	Mean Inhibition Rate ± SD (%)
DHA	Control	0.00 ± 1.75
	PC	76.36 ± 0.06
	6.25 μM	0.14 ± 0.11
	12.5 μM	0.24 ± 0.07
	25 μM	3.96 ± 0.24
	50 μM	4.60 ± 0.16
	100 μM	16.39 ± 1.00 *

Control: phosphate-buffered saline; PC: 1% aminoguanidine hydrochloride; Compared with control group, * *p* < 0.01.

**Table 2:** Reaction system of AGEs inhibition test.

	Volume/mL	A	B	C	D
Name					
The glycation reaction solution (2×)		1	0	1	0
Sample/positive control solution		1	1	0	0
PBS/sample solvent		0	1	1	2

A—the fluorescence intensity of sample; B—the fluorescence intensity of sample solvent; C—the fluorescence intensity of glycation system; D—the fluorescence intensity of the solvent of glycation system.

## Data Availability

All data were within the manuscript.

## Abbreviations

The following abbreviations are used in this manuscript:

DHA	dihydroartemisinin
HDF	human dermal fibroblast
DPC	dermal papilla cells
OCT	octopirox
PC	positive control group
NC	negative control group
BC	blank control group
AGEs	advanced glycation end products

## References

[1] Kamila M.Z.-P., Helena R. (2020). The Effectiveness of Ferulic Acid and Microneedling in Reducing Signs of Photoaging: A Split-Face Comparative Study. Dermatol. Ther..

[2] Nagoor Meeran M.F., Javed H., Al Taee H., Azimullah S., Ojha S.K. (2017). Pharmacological Properties and Molecular Mechanisms of Thymol: Prospects for Its Therapeutic Potential and Pharmaceutical Development. Front. Pharmacol..

[3] Dhaliwal S., Rybak I., Pourang A., Burney W., Haas K., Sandhu S., Crawford R., Sivamani R.K. (2021). Randomized Double-Blind Vehicle Controlled Study of the Effects of Topical Acetyl Zingerone on Photoaging. J. Cosmet. Dermatol..

[4] Bai X.-Y., Liu P., Chai Y.-W., Wang Y., Ren S.-H., Li Y.-Y., Zhou H. (2020). Artesunate Attenuates 2, 4-Dinitrochlorobenzene-Induced Atopic Dermatitis by down-Regulating Th17 Cell Responses in BALB/c Mice. Eur. J. Pharmacol..

[5] Wang G.-J., Gao X.-Y., Wu Y., He H.-Q., Yu Y., Qin H.-H., Shen W.-T. (2019). Evaluation of the Efficacy and Tolerance of Artemether Emulsion for the Treatment of Papulopustular Rosacea: A Randomized Pilot Study. J. Dermatol. Treat..

[6] Härtel A., Jung T., Sift Carter R. (2018). Artemether for Topical Use in Patients with Seborrhoeic Keratosis. Br. J. Dermatol..

[7] Tian L., Ke D., Hong Y., Zhang C., Tian D., Chen L., Zhan L., Zong S. (2021). Artesunate Treatment Ameliorates Ultraviolet Irradiation-Driven Skin Photoaging via Increasing β-Catenin Expression. Aging.

[8] Efferth T., Oesch F. (2021). The Immunosuppressive Activity of Artemisinin-Type Drugs towards Inflammatory and Autoimmune Diseases. Med. Res. Rev..

[9] Qin H., Zhu X., Liu X., Wang Y., Liang J., Wu H., Wu J. (2025). Dihydroartemisinin Alleviates the Symptoms of a Mouse Model of Systemic Lupus Erythematosus through Regulating Splenic T/B-Cell Heterogeneity. Curr. Issues Mol. Biol..

[10] Xue X., Dong Z., Deng Y., Yin S., Wang P., Liao Y., Hu G., Chen Y. (2020). Dihydroartemisinin alleviates atopic dermatitis in mice by inhibiting mast cell infiltration. J. South. Med. Univ..

[11] Yu R., Jin L., Li F., Fujimoto M., Wei Q., Lin Z., Ren X., Jin Q., Li H., Meng F. (2020). Dihydroartemisinin Inhibits Melanoma by Regulating CTL/Treg Anti-Tumor Immunity and STAT3-Mediated Apoptosis via IL-10 Dependent Manner. J. Dermatol. Sci..

[12] Ekiert H., Klimek-Szczykutowicz M., Rzepiela A., Klin P., Szopa A. (2022). Artemisia Species with High Biological Values as a Potential Source of Medicinal and Cosmetic Raw Materials. Molecules.

[13] Zhao Y., Zhu L., Yang L., Chen M., Sun P., Ma Y., Zhang D., Zhao Y., Jia H. (2024). In Vitro and in Vivo Anti-Eczema Effect of Artemisia Annua Aqueous Extract and Its Component Profiling. J. Ethnopharmacol..

[14] Maxim C., Badeanu M., Turcov D., Suteu D. (2025). Potential of Artemisia Annua Hydroalcoholic Extracts in Skin Care and Dermatocosmetic Products. Not. Bot. Horti Agrobot. Cluj-Napoca.

[15] Weathers P., Towler M., Kiani B.H., Dolivo D., Dominko T. (2024). Differential Anti-Fibrotic and Remodeling Responses of Human Dermal Fibroblasts to Artemisia Sp., Artemisinin, and Its Derivatives. Molecules.

[16] Liu J.-M., Jin Q.-X., Fujimoto M., Li F.-F., Jin L.-B., Yu R., Yan G.-H., Zhu L.-H., Meng F.-P., Zhang Q.-G. (2021). Dihydroartemisinin Alleviates Imiquimod-Induced Psoriasis-like Skin Lesion in Mice Involving Modulation of IL-23/Th17 Axis. Front. Pharmacol..

[17] Xu W., Zhu Q., Chen J., He J., Yuan A., Cao P., Zhang L. (2025). Exploring the Mechanisms of Artemisinin and Its Derivatives in the Treatment of Atopic Dermatitis Based on Network Pharmacology and Molecular Docking: A Review. Medicine.

[18] Reilly D.M., Lozano J. (2021). Skin Collagen through the Lifestages: Importance for Skin Health and Beauty. Plast. Aesthetic Res..

[19] Chen S.J., Yuan W., Mori Y., Levenson A., Trojanowska M., Varga J. (1999). Stimulation of Type I Collagen Transcription in Human Skin Fibroblasts by TGF-Beta: Involvement of Smad 3. J. Investig. Dermatol..

[20] Yang S., Wang X., Xiao W., Xu Z., Ye H., Sha X., Yang H. (2022). Dihydroartemisinin Exerts Antifibrotic and Anti-Inflammatory Effects in Graves’ Ophthalmopathy by Targeting Orbital Fibroblasts. Front. Endocrinol..

[21] Ding Y.-W., Li Y., Zhang Z.-W., Dao J.-W., Wei D.-X. (2024). Hydrogel Forming Microneedles Loaded with VEGF and Ritlecitinib/Polyhydroxyalkanoates Nanoparticles for Mini-Invasive Androgenetic Alopecia Treatment. Bioact. Mater..

[22] Papukashvili D., Rcheulishvili N., Liu C., Xie F., Tyagi D., He Y., Wang P.G. (2021). Perspectives on miRNAs Targeting DKK1 for Developing Hair Regeneration Therapy. Cells.

[23] Shin D.W. (2022). The Molecular Mechanism of Natural Products Activating Wnt/β-Catenin Signaling Pathway for Improving Hair Loss. Life.

[24] Harada K., Saito M., Sugita T., Tsuboi R. (2015). Malassezia Species and Their Associated Skin Diseases. J. Dermatol..

[25] Saunte D.M.L., Gaitanis G., Hay R.J. (2020). Malassezia-Associated Skin Diseases, the Use of Diagnostics and Treatment. Front. Cell. Infect. Microbiol..

[26] Liang X., Chen D., Wang J., Liao B., Shen J., Ye X., Wang Z., Zhu C., Gou L., Zhou X. (2023). Artemisinins Inhibit Oral Candidiasis Caused by Candida Albicans through the Repression on Its Hyphal Development. Int. J. Oral Sci..

[27] Moore C.M., Hoey E.M., Trudgett A., Timson D.J. (2011). Artemisinins Act through at Least Two Targets in a Yeast Model. FEMS Yeast Res..

[28] Costa E.F., Magalhães W.V., Di Stasi L.C. (2022). Recent Advances in Herbal-Derived Products with Skin Anti-Aging Properties and Cosmetic Applications. Molecules.

[29] Solano F. (2014). Melanins: Skin Pigments and Much More—Types, Structural Models, Biological Functions, and Formation Routes. New J. Sci..

[30] Pan C., Liu X., Zheng Y., Zhang Z., Li Y., Che B., Liu G., Zhang L., Dong C., Aisa H.A. (2023). The Mechanisms of Melanogenesis Inhibition by Glabridin: Molecular Docking, PKA/MITF and MAPK/MITF Pathways. Food Sci. Hum. Wellness.

[31] Liu E., Bai S., Huang Y., Pang Y., Zhang X., Zeng J., Guo J. (2025). Studies on the Critical Therapeutic Role of Artemisinin and Its Derivatives in Melanoma: A Review of Preclinical Evidence. Curr. Treat. Options Oncol..

[32] Chen C., Zhang J.-Q., Li L., Guo M., He Y., Dong Y., Meng H., Yi F. (2022). Advanced Glycation End Products in the Skin: Molecular Mechanisms, Methods of Measurement, and Inhibitory Pathways. Front. Med..

[33] Reddy V.P., Aryal P., Darkwah E.K. (2022). Advanced Glycation End Products in Health and Disease. Microorganisms.

[34] El Hosry L., Elias V., Chamoun V., Halawi M., Cayot P., Nehme A., Bou-Maroun E. (2025). Maillard Reaction: Mechanism, Influencing Parameters, Advantages, Disadvantages, and Food Industrial Applications: A Review. Foods.

